# Low-field magnetic resonance imaging offers potential for measuring tibial component migration

**DOI:** 10.1186/s40634-017-0116-2

**Published:** 2018-01-12

**Authors:** F. F. Schröder, N. J. J. Verdonschot, B. ten Haken, A. Peters, A. J. H. Vochteloo, D. F. M. Pakvis, R. Huis in’t Veld

**Affiliations:** 1 Centre for Orthopaedic Surgery OCON, Hengelo, The Netherlands; 2 MIRA Institute for Biomedical Technology and Technical Medicine, University of Twente, Enschede, the Netherlands; 3 Radboud Institute for Health Sciences, Orthopaedic Research Lab, Radboud university medical center, Nijmegen, The Netherlands

**Keywords:** Prosthetic loosening, Precision, Low-field MRI, Roentgen stereophotogrammetric analysis (RSA), Migration

## Abstract

**Background:**

Roentgen stereophotogrammetric analysis (RSA) is used to measure early prosthetic migration and to predict future implant failure. RSA has several disadvantages, such as the need for perioperatively inserted tantalum markers. Therefore, this study evaluates low-field MRI as an alternative to RSA. The use of traditional MRI with prostheses induces disturbing metal artifacts which are reduced by low-field MRI. The purpose of this study is to assess the feasibility to use low-field (0.25 Tesla) MRI for measuring the precision of zero motion. This was assessed by calculating the virtual prosthetic motion of a zero-motion prosthetic reconstruction in multiple scanning sessions. Furthermore, the effects of different registration methods on these virtual motions were tested.

**Results:**

The precision of zero motion for low-field MRI was between 0.584 mm and 1.974 mm for translation and 0.884° and 3.774° for rotation. The manual registration method seemed most accurate, with μ ≤ 0.13 mm (σ ≤ 0.931 mm) for translation and μ ≤ 0.15° (σ ≤ 1.63°) for rotation.

**Conclusion:**

Low-field MRI is not yet as precise as today’s golden standard (marker based RSA) as reported in the literature. However, low-field MRI is feasible of measuring the relative position of bone and implant with comparable precision as obtained with marker-free RSA techniques. Of the three registration methods tested, manual registration was most accurate. Before starting clinical validation further research is necessary and should focus on improving scan sequences and registration algorithms.

## Background

Early prosthetic migration is associated with future aseptic loosening. (Valstar et al. 2002) Roentgen stereophotogrammetric analysis (RSA) is the golden standard in measuring early component migration.(Kärrholm et al. 1997; Vrooman et al. 1998; Kärrholm et al. 2006) Currently, the clinically obtained accuracy of conventional RSA varies between 0.05 and 0.5 mm for translation and 0.15° to 1.15° for rotation (95% confidence intervals (CI)). This accuracy level is considered clinically relevant for diagnosing early prosthetic migration. (Valstar et al. 2002; Seehaus et al. 2012).

Clinical application of the RSA technique is limited because of the extended operation time due to the requirement of perioperative insertion of tantalum markers, the use of calibration cages and specific radiological facilities with two X-ray machines, the availability of specialized software and trained personnel, and the fact that patients are exposed to additional radiation during longitudinal RSA studies.(Kaptein et al. 2003; Otten et al. 2017).

Improvements have focused on “marker-free” RSA methods. However, these are less accurate when compared to conventional RSA, and additional CT models are needed.(de Bruin et al. 2008; Seehaus et al. 2012) Previous attempts to use MRI models instead of CT have failed, because MRI models interfere with the used X-ray shape-matching procedure which is based on Hounsfield units.(Moro-oka et al. 2007).

**Fig. 1:**
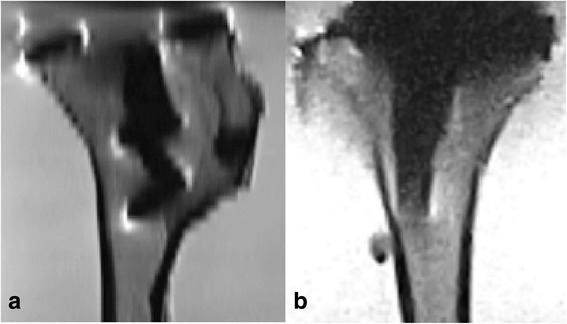
**a** MRI slices of the tibial component of a total knee prosthesis made with high field MRI with a TSE PD sequence in the sagittal direction. **b** MRI slices of the tibial component of a total knee prosthesis made with low-field MRI made with a TSE/FSE PD sequence in the sagittal direction

MRI has some characteristics that make it less suitable for bone and prosthetic imaging: it provides lower bone contrast than CT; it suffers from spatial and geometric distortions, field inhomogeneity, and metal artefacts.(Doran et al. 2005; Vandevenne et al. 2007; Moro-oka et al. 2007) (Fig. 1a) Disadvantages of MRI may be partly overcome by the use of low-field MRI. (Fig. 1b) A lower magnetic field reduces spatial and geometric distortions, increases the field homogeneity and bone contrast, and decreases metal artefacts.(Ghazinoor et al. 2007) Furthermore, MRI offers imaging of soft tissues, which provides clinicians with additional diagnostic information. Although low-field MRI (< 0.5 T) is rarely used in clinical practice, it is considered to be highly suitable for musculoskeletal imaging. (Ghazinoor et al. 2007; Lee et al. 2013).

In order to determine the potential of low-field MRI as an alternative, its precision in measuring position of implant and bone must be calculated. The precision of low-field MRI for measuring zero motion depends partly on the imaging technique and partly on the analysis process, which consists of segmentation and registration. It is important to quantify the precision of low-field MRI first, since this is prerequisite before implementing the method in clinical practice.

Segmentation can be performed manually or (semi-)automatically and provides 3D models to be used in subsequent registration steps. In order to calculate prosthetic migration, model matching which is called registration between 3D models is necessary.

An accurate, fully automatic segmentation and registration procedure is more standardized and time-effective than manual registration. However, manual registration may be more precise and visual feed-back can be interpreted while performing the segmentations steps. In order to determine which registration method is most suitable three methods: manual, semi-automatic with the use of 3D reference models and semi-automatic without the use of 3D reference models were compared.

The primary goal of this study was to assess the feasibility to use low-field (0.25 Tesla) MRI for measuring the precision of zero motion, using a tibial component of a total knee prosthesis in a phantom.

Additionally, the type of registration method most suitable for measuring the position of the tibial component was assessed.

## Method

**Fig. 2:**
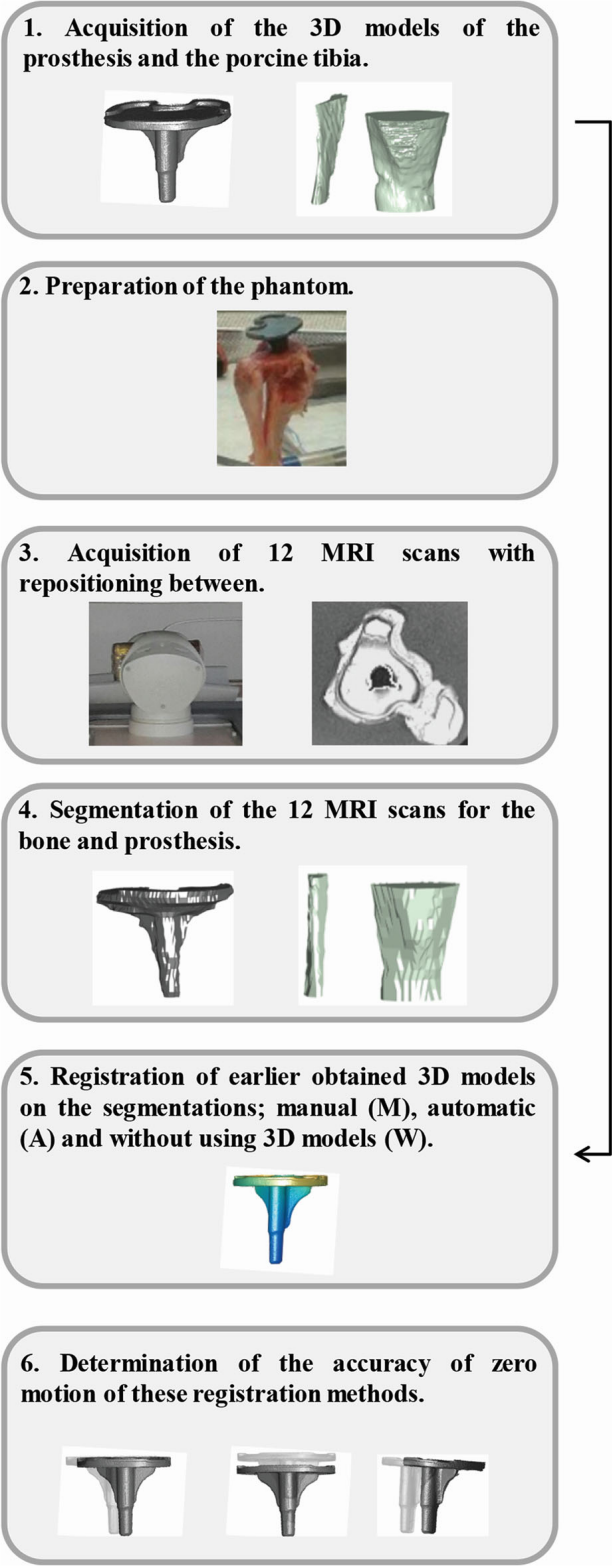
Study design

The aim of the following study was to determine the feasibility of low-field MRI to measure the precision of zero motion, several steps were taken (Fig. 2).

### Creating 3D reference models

In order to determine the position of the tibial component (Genesis II, Smith & Nephew Inc. Memphis USA) with respect to the surrounding bone, 3D geometrical models of both the prosthesis and the bone were made. The study focused on the tibial component of a total knee arthroplasty because according to the Swedish arthroplasty register their failure of the tibial component (6.8%) is much more frequent than that of the femoral component (1.1%).(Robertsson Otto et al. 2017) The outer surface of the prosthesis was created using a 3D optical scanner (Konica Minolta Vivid 910). The 3D model of the porcine tibial bone was created by scanning the bone (without containing the implant) on a low-field MRI (0.25 T) (Esaote G-scan brio) with a 3D SHARC sequence (TR/TE 25/12.5, slice thickness 0.4 mm, acquisition time 5:38, field of view 200 mm with a matrix of 512 × 512). In order to obtain the 3D surface model of the bone, an imaging expert semi-automatically segmented the cortical bone of both the tibia and fibula with Mimics (Mimics Research 18.0, Materialise NV), resulting in an inner and outer surface of the porcine tibial bone just distal from the site where the future prosthesis would be positioned.

### MRI acquisitions

Subsequently, a phantom was created by implanting the tibial component of the knee prosthesis into a porcine tibial bone and placing this bone in a gelatine solution (2%). Gelatine was chosen for its properties such as relaxation time and elasticity in order to mimic the soft tissue of the knee.(Madsen et al. 1991).

In this phantom study, it was ensured that the prosthesis did not migrate with respect to the surrounding bone (zero motion) during the various scanning sessions. The phantom was relocated 12 times, the relocation varied between maximal 25 degrees rotation left and right from the supine position. The differences in translation and rotations of the tibial plate with respect to the position of the bone measured across the 12 acquisitions were defined as the precision of zero-motion for low-field MRI. Similarly as described in ISO guideline for RSA studies.(ISO 16087 2013) During each of the 12 acquisitions, the phantom was scanned in a transverse direction on a low-field MRI with a 2D PD-weighted metal artifact reduction sequence (MARS) PD-XMAR (TR/TE 1020/10, slice thickness 3 mm, acquisition time 5:08 min, field of view 180 mm with a matrix of 224 × 224). This particular sequence was chosen because it reduces metal artefacts, provides good contrast between bone and surrounding soft tissue, and is capable of adequately imaging the human knee. During all acquisitions, the temperatures of the room and the phantom were kept constant (21 degrees Centigrade).

### Segmentation and 3D model reconstruction

The prosthesis and the cortical bone of the tibia and fibula distal to the prosthesis on the 12 MR-acquisitions obtained were semi-automatically segmented by an imaging expert in approximate 30 min per acquisition using Mimics. The segmentations included the following steps:

1. A suitable threshold was determined by applying a profile line in Mimics.
2. A region growing algorithm was applied to collect the connected voxels.
3. Abundant voxels were erased manually.
4. Missing voxels in the segmented region were filled with the morphology operation (“closing”).
5. All slices were checked manually, abundant voxels were erased, and missing voxels were filled.
6. A 3D model (the segmentation model) was constructed from the connected voxels.

This resulted in 12 segmentation models of the bone and 12 of the prosthesis.

### Analysis

The segmentation models of the bone and prosthesis were registered to the 3D reference models constructed earlier. Registration was performed to transform all segmented datasets into the reference coordinate system in order to facilitate future calculations. Registration was performed in three different ways in order to determine which method is most accurate. The three methods are described below.

1. Marker-free MRI manual registration (MMRI-M): The segmentation of the bone was registered to the 3D reference model of the bone with the automatic registration algorithm available in Mimics (global registration followed by local registration). The 3D reference model of the prosthesis was manually fit to the segmentation of the prosthesis by an imaging expert in approximately 5 min. Several landmarks of the prosthetic model, e.g. the posterior edge and the distal notch, were precisely matched on the segmentation.
2. Marker-free MRI automatic registration, fully automatic registration with the use of 3D reference models (MMRI-A): The automatic registration algorithm available in Mimics was used to register the prosthetic and bone segmentations to the 3D reference models of the prosthetic and the bone.
3. Marker-free MRI automatic registration, fully automatic without the use of 3D reference models (MMRI-W): The segmentations of the prosthesis and the bone based on the MRI scans of acquisition number one were taken as a reference model instead of the 3D reference models. Using the automatic registration algorithm available in Mimics, these were registered to the segmentations of the prosthesis and bone based on the remaining acquisitions.

During registration, all 3D segmentations were matched to a 3D reference model and transformed to the reference coordinate system. Subsequently, using a procrustes algorithm in Matlab (R2015b, Mathworks©), the position (3-D translations and rotations) of the prosthesis with respect to the bone was calculated across two acquisitions (one with two, two with three, etc.) with all three registration methods enlisted before.

Results are presented for all three registration methods. The translation and rotation for all six degrees of freedom of the prosthesis calculated from the midpoint are presented for the 12 acquisitions, with the mean (μ) and standard deviation (σ), which defined the precision of zero motion. In order to compare the different registration algorithms, three distance plot presenting the migration of the tibial component calculated par point were compiled. Boxplots were constructed to calculate the precision of zero motion per degree of freedom per registration method at 95% CI; μ ± 1.96 σ. The boxplots also visualize the current golden standard, i.e. 0.5 mm for translation and 1.15° for rotation (95% CI)(Valstar et al. 2002; Seehaus et al. 2012).

## Results

In Table 1, each row represents a calculated zero-motion result, which indicates the difference between two acquisitions for each of the three registration methods applied (MMRI-M, MMRI-A, and MMRI-W). Of the three registration methods, MMRI-M measured the precision of zero motion most precise, with a maximal mean error of 0.128 mm for translation (maximal σ 0.931 mm) and of 0.152° for rotation (maximal σ 1.630°). External internal migration was fully within the range considered clinically relevant. For MMRI-A and MMRI-W, maximal errors for translation and rotation were 0.147 mm for translation (σ 1.974 mm) and 0.033° for rotation (σ 3.774°); and 0.136 mm for translation (σ 1.518 mm) and 0.068° for rotation (σ 2.527°), respectively.

**Table 1:** MRI images of the phantom of 12 different acquisitions have been registered with three different methods

Acquisition (Reference acquisition)	medial-lateral migration(mm)	distal-proximal migration(mm)	posterior-anterior migration(mm)	flexion-extension migration (deg)	external-internal migration (deg)	varus-valgus migration (deg)
MMRI-M						
1 (2)	−0.367	1.657	0.129	−1.460	0.984	0.557
2 (3)	0.241	−1.313	−0.446	0.682	0.681	1.846
3 (4)	−0.158	0.711	−0.535	0.829	−0.107	−0.960
4 (5)	0.857	−0.209	0.359	−1.183	0.386	−0.952
5 (6)	−0.258	−0.116	−0.424	1.623	−0.080	2.125
6 (7)	0.912	0.426	−0.428	2.272	1.039	−0.748
7 (8)	−0.724	−0.525	0.885	−3.657	−0.109	−1.624
8 (9)	−0.146	1.334	−0.699	0.518	0.570	0.456
9 (10)	−0.084	−1.207	−0.295	−1.197	−1.780	−0.821
10 (11)	1.231	−0.372	0.985	0.775	0.712	0.291
11 (12)	0.135	0.516	−0.118	0.465	0.781	0.301
12 (1)	−0.105	−0.642	−0.833	0.939	−1.252	−0.398
μ(σ)	0.128 (0.584)	0.022 (0.931)	−0.118 (0.593)	0.050 (1.630)	0.152 (0.884)	0.006 (1.147)
MMRI-A						
1 (2)	0.288	−0.561	1.836	1.506	0.936	−1.022
2 (3)	1.571	−2.674	−2.178	2.964	0.900	6.187
3 (4)	−0.738	3.648	0.381	−2.857	0.298	−3.799
4 (5)	−0.040	−2.650	−0.001	0.952	−0.840	1.247
5 (6)	−1.006	−1.651	−1.670	−4.091	−0.321	3.394
6 (7)	1.210	0.226	1.094	1.968	1.573	−3.264
7 (8)	1.489	0.986	0.597	2.472	1.206	−1.856
8 (9)	−2.071	0.131	−0.667	−5.606	−1.682	0.943
9 (10)	−0.568	2.232	−0.203	1.584	−4.138	2.737
10 (11)	0.535	0.079	0.495	0.628	1.160	−0.290
11 (12)	1.565	−1.566	−1.880	2.888	0.125	3.453
12 (1)	−0.470	1.964	1.167	−2.522	0.393	−7.439
μ(σ)	0.147 (1.172)	0.014 (1.974)	−0.086 (1.284)	−0.010 (2.952)	−0.033 (1.595)	0.024 (3.774)
MMRI-W						
1 (2)	−0.326	−0.173	1.702	1.725	−0.779	−0.735
2 (3)	1.315	−0.736	−1.071	−0.359	1.727	0.665
3 (4)	0.094	0.356	−0.081	2.417	0.516	−0.291
4 (5)	0.467	−0.804	0.716	−0.789	−0.416	0.502
5 (6)	−0.432	2.489	−1.456	−2.723	−0.360	−0.305
6 (7)	1.229	−3.267	0.182	4.319	0.750	0.416
7 (8)	0.196	0.690	−0.240	−2.665	0.295	0.291
8 (9)	0.369	1.619	−0.062	2.500	0.206	−0.068
9 (10)	−2.113	1.378	0.618	−3.284	−2.317	−1.114
10 (11)	0.577	−1.102	−0.718	0.452	0.553	0.383
11 (12)	1.669	0.119	−1.792	1.847	1.364	2.007
12 (1)	−1.414	−0.956	0.746	−2.623	−1.781	−1.790
μ(σ)	0.136 (1.100)	−0.032 (1.518)	−0.121 (1.012)	0.068 (2.527)	−0.020 (1.189)	−0.003 (0.967)

This table shows the migration calculated using the MRI data of each acquisition with respect to the previous acquisition

A perfect registration result would be zero migration

**Fig. 3:**
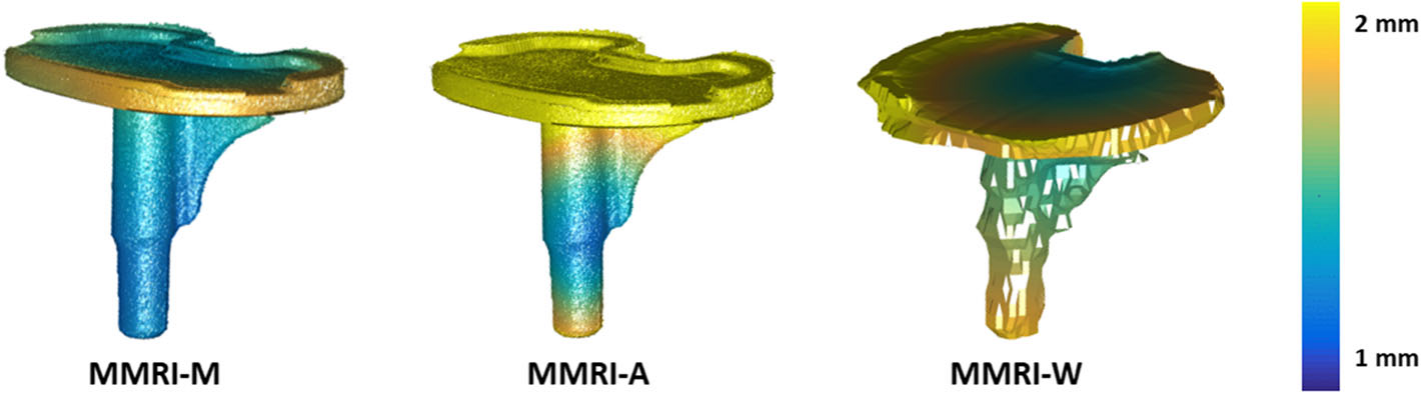
Distance plots for translation of the prosthesis with respect to the bone for the three types of registration methods used. (MMRI-M, MMRI-A and MMRI-W)

Figure 3 shows the distance plots of the tibial component. In this figure, the difference between the calculated value (error) and the real migration (zero) is visualized on the surface of the tibial component. The smallest error was seen in the distal part of the stem of the tibial component. Of the proximal plate, the posterior area had the smallest error.

**Fig. 4:**
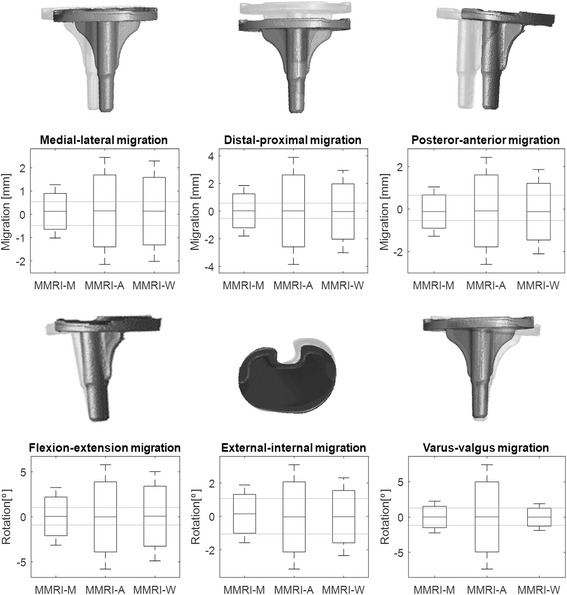
Boxplots for the six degrees of freedom, three for translation and three for rotation, for MMRI-M, MMRI-A and MMRI-W (95% CI; μ ± 2σ). The dashed horizontal lines indicate bounding range of RSA accuracy reported in literature

The boxplots in Fig. 4 display the calculated values per registration method with the ranges of the golden standard. As is evident, the results are mostly out of range for all degrees of freedom, regardless of the registration method used. The distal-proximal direction shows the largest translation error.

## Discussion

The most important finding of the present study is that the low-field MRI method as utilized in this study is not yet as precise as the golden standard RSA. However, low-field MRI is feasible of measuring the relative position of bone and implant with an error of μ ≤ 0.13 mm (σ ≤ 0.931 mm) for translation and μ ≤ 0.15° (σ ≤ 1.63°) for rotation when the manual registration algorithm is used, which indicates that with some improvements this technique could reach adequate precision.

### Precision of zero motion

The values for the precision of zero motion for rotation are more precise than those in recent marker-free RSA studies from Seehaus et al. (Seehaus et al. 2012) μ ≤ 1.64° (σ ≤ 3.17°) and de Bruin et al. (de Bruin et al. 2008) μ ≤ 0.21° (σ ≤ 3.26°). However, in these studies, the values obtained for translation were comparable (μ ≤ −0.363 mm (σ ≤ 0.876 mm)) and more accurate (μ ≤ −0.083 mm (σ ≤ 0.295 mm)) than the values obtained in this study. In this study, the largest measurement error for translation is in the distal-proximal direction. An explanation for this is that only 2D metal artefact reducing sequences were available on the low-field MRI system, which resulted in a 3 mm through-plane resolution in the distal proximal direction compared to a resolution of 0.4 mm in the medial-lateral and posterior-anterior direction. An improvement in the through-plane resolution is expected to result in a more detailed segmentation and thereby to contribute to a smaller standard deviation for translation. To have a similar through-plane resolution in all directions further research should focus on improving 3D sequences. Another option which could reduce the measurement error in the proximal-distal direction is by changing the scan direction to sagittal or coronal.

Nevertheless, neither the results obtained in this study nor those from marker-free RSA research are as accurate as the golden standard. (Valstar et al. 2002; Seehaus et al. 2012).

To reflect on the precision of the low-field MRI method, reference values for RSA were obtained from the literature. It should be noted that these reported values are subject to variations of the exact methods and implants used. These reference values should therefore be used with caution and can only serve as an indication of the ‘overall precision’ of marker based and marker-free RSA techniques.

### Registration method

Marker-free MRI with manual registration (MMRI-M) had the smallest registration error. Manually, it was possible to match according to specific landmarks such as the posterior notch of the proximal plate or on the distal stem, which explains the favourable results. However, contrary to automatic registration, manual registration is susceptible to observer variation. It is also more time-consuming.

Despite its lower accuracy, the registration method that does not use a 3D reference model (MMRI-W) remains interesting. MMRI-W is an automatic method and is not susceptible to observer variation. Because it omits reference to a 3D reference model, the MMRI-W method makes it possible to determine the position of any prosthesis implanted in a patient, and would thus be the most accessible method when applied in daily practice. This makes it worthwhile to work on improvements of this method which is a somewhat less accurate automatic registration method.

### Strengths and limitations of the technique and the study

In addition to its potential to measure prosthetic migration, low-field MRI is excellently suited for judging the soft tissue structures surrounding the implants. Future research should investigate the added clinical benefit of being able to assess soft tissue structures as well as prosthetic positioning and migration. If low-field MRI is capable of these combined evaluations, the technique could be beneficial for individual patients who have recurrent or persisting symptoms after total knee arthroplasty. Low-field MRI could aid to diagnose whether this is caused by migration of the prosthesis and/or by other issues such as malpositioning or soft tissue impingement problems. Furthermore, it should be noted that compared to high-field MRI, low-field MRI is considerably less expensive and could therefore be a relatively cost-effective manner to assess soft tissue aspects as well as prosthetic migration.

Obviously, this study has some limitations. Firstly, since it is a feasibility study, only one porcine tibia with a tibial component without insert and femoral component was used, and results were analysed by one imaging specialist. For further validation more subjects should be analysed by more than one imaging specialist. An additional shortcoming is that a phantom will always be a limited representation of the human knee. In this study a gelation solution was used to mimic the soft tissue. (Madsen et al. 1991) Gelatine is a more homogeneous substance than human tissue, and while it does mimic globally soft tissue imaging properties, it lacks the variety of soft tissues in the human knee. If the low-field MRI’s accuracy is tested in the human knee, the knee’s reduced homogeneity will influence the size of the metal artifacts, which could affect the accuracy of the measurements. Moreover, the phantom was at room temperature (21 °C). The higher temperature of the human knee could also affect the image quality (Petrén-mallmin marianne et al., 1993).

Secondly, during the acquisitions available sequences on the low-field MRI system, and during the automatic registration procedure the registration algorithm available in Mimics were used. Although these methods can be considered as state of the art, further improvements on these aspects can be made in order to further reduce the registration errors. From these two aspects, it is proposed to first focus on improving the MRI sequences on the low-field system. When metal artefacts are reduced even more, segmentations become more similar and registration more accurate. Subsequently, research could focus on further improving the registration methods. Other registration options such as rigid image registration techniques should be further explored. This method allows scans to be directly registered to each other, without the necessity of any segmentation.(Vandemeulebroucke et al. 2013).

Thirdly, this study focuses on the accuracy of zero motion of low-field MRI. For further validation, it is necessary to generate true migrations with a micromanipulator in order to evaluate low-field MRI’s ability to measure prosthetic migration in a multiple human cadaver study with a total knee prosthesis. This should be followed by a clinical validation. If low-field MRI is used in clinical practice movement artifacts may occur. In today’s practice, patients are instructed before they are scanned in a high-field MRI scanner to minimize movement artefacts. Since the low-field MRI protocol for measuring migration is shorter than a clinical high-field MRI we expect that when the patient is well instructed movement artefacts can be negligible.

## Conclusions

In conclusion low-field MRI as utilized in this study appeared not yet to be as precise as the golden standard RSA. However, RSA has a history of over 50 years. Interestingly, results of the present study showed that low-field MRI is feasible of measuring the relative position of bone and implant with a precision which is comparable to marker-free RSA techniques.

Of the three tested registration methods, manual registration was most accurate. However, manual registration is susceptible to observer variation and is more time-consuming.

Further research is necessary and should focus on improving scan sequences and registration algorithms, in order to further improve the precision and thereby working towards clinical validation. Consequently, once this technique is validated within a patient cohort, low-field MRI is suggested to be a marker free and radiation free alternative for RSA.

## Abbreviations

MMRI-A: Marker-free automatic registration
MMRI-M: Marker-free manual registration
MMRI-W: Marker-free automatic registration without using 3D reference models
RSA: Roentgen stereophotogrammetric analysis
μ: Mean
σ: Standard deviation

## References

[CR1] de Bruin PW, Kaptein BL, Stoel BC (2008). Image-based RSA: roentgen stereophotogrammetric analysis based on 2D-3D image registration. J Biomech.

[CR2] Doran SJ, Charles-Edwards L, S a R, Leach MO (2005). A complete distortion correction for MR images: I. Gradient warp correction. Phys Med Biol.

[CR3] Ghazinoor S, Crues JV, Crowley C (2007). Low-field musculoskeletal MRI. J Magn Reson Imaging.

[CR4] ISO 16087 (2013) Implants for surgery — Roentgen stereophotogrammetric analysis for the assessment of migration of orthopaedic implants. https://www.iso.org/obp/ui/#iso:std:iso:16087:ed-1:v1:en

[CR5] Kaptein BL, Valstar ER, Stoel BC (2003). A new model-based RSA method validated using CAD models and models from reversed engineering. J Biomech.

[CR6] Kärrholm J, Gill RHS, Valstar ER (2006). The history and future of Radiostereometric analysis. Clin Orthop Relat Res.

[CR7] Kärrholm J, Herberts P, Hultmark P (1997). Radiostereometry of hip prostheses. Clin Orthop Relat Res.

[CR8] Lee CS, Davis SM, McGroder C (2013). Analysis of low-field magnetic resonance imaging scanners for evaluation of knee pathology based on arthroscopy. Orthop J Sport Med.

[CR9] Madsen EL, Blechinger JC, Frank GR (1991). Low-contrast focal lesion detectability phantom for 1 H MR imaging. Med Phys.

[CR10] Moro-oka T, Hamai S, Miura H (2007). Can magnetic resonance imaging–derived bone models be used for accurate motion measurement with single-plane three-dimensional shape registration?. J Orthop Res.

[CR11] Otten V, Maguire GQ, Noz ME (2017). Are CT scans a satisfactory substitute for the follow-up of RSA migration studies of Uncemented cups? A comparison of RSA double examinations and CT datasets of 46 Total hip Arthroplasties. Biomed Res Int.

[CR12] Ericsson A, Rauschning W, Hemmingsson A, Petrén-mallmin marianne (1993). The effect of temperature on MR relaxation times and signal intensities for human tissues. MAGMA.

[CR13] Robertsson O, Annette W-D, Lars L, Martin S (2017) Annual Report 2017, Swedisch knee arthroplasty register., 2017th edn. Lund. http://myknee.se/pdf/SVK_2017_Eng_1.0.pdf

[CR14] Seehaus F, Olender GD, Kaptein BL (2012). Markerless roentgen Stereophotogrammetric analysis for in vivo implant migration measurement using three dimensional surface models to represent bone. J Biomech.

[CR15] Valstar ER, Nelissen RGHH, Reiber JHC, Rozing PM (2002). The use of roentgen stereophotogrammetry to study micromotion of orthopaedic implants. ISPRS J Photogramm Remote Sens.

[CR16] Vandemeulebroucke J, Deklerck R, Temmermans F (2013). Automated estimation of hip prosthesis migration: a feasibility study. Tescher AG (ed) proceedings of SPIE - the International Society for Optical Engineering.

[CR17] Vandevenne JE, Vanhoenacker FM, Parizel PM et al (2007) Reduction of metal artefacts in musculoskeletal MR imaging. JBR-BTR 90:345–349. https://www.researchgate.net/publication/230728034_Reduction_of_metal_artifacts_in_musculoskeletal_MR_imaging 18085188

[CR18] Vrooman HA, Valstar ER, Brand G-J (1998). Fast and accurate automated measurements in digitized stereophotogrammetric radiographs. J Biomech.

